# An Innovative Minimally Invasive Delta Fixation for Thoracolumbar Fracture With Diffuse Idiopathic Skeletal Hyperostosis

**DOI:** 10.7759/cureus.77216

**Published:** 2025-01-10

**Authors:** Angel Oscar Paz Flores, Masato Tanaka, Christian Heng, Shinya Arataki, Tadashi Komatsubara

**Affiliations:** 1 Department of Orthopedic Surgery, Okayama Rosai Hospital, Okayama, JPN

**Keywords:** c-arm-free, diffuse idiopathic skeletal hyperostosis, minimally invasive surgery, navigation, thoracolumbar fracture

## Abstract

Diffuse idiopathic skeletal hyperostosis (DISH) is a relatively common disease for elderly people, characterized by a tendency of ossification of ligaments and tendons. Spinal fracture in patients with diffuse idiopathic skeletal hyperostosis is a very unstable fracture and usually needs posterior long spinal fixation. Conventional pedicle screw fixation is the standard method for these ankylosed spines. However, with the traditional technique, screw loosening, screw pullout, implant failure, and nonunion are often encountered due to the osteoporotic bone. Recently, transdiscal screw fixation for this fracture was reported as a strong anchor for the osteoporotic spine. Furthermore, triangular fixation, which is the combination of transdiscal screws and downward screws, has reported excellent results for long posterior spinal fusion for adult spinal deformity. We present an 86-year-old male with severe low back pain due to an L1 fracture treated with a novel minimally invasive technique via delta fixation technique.

## Introduction

Diffuse idiopathic skeletal hyperostosis (DISH) is relatively common in older people and usually causes only stiffness of the spinal column, with no severe symptoms. However, if spinal fractures occur in DISH patients, early diagnosis and treatment of those fractures are very important. Because DISH fractures are very unstable due to the long lever arms of the fused segments, treatment delay may cause serious consequences. Furthermore, those fractures have a high risk of spinal cord injury (approximately 30%) [1]. Unfortunately, >50% of spinal cord injury cases are due to the delay of the doctor's correct diagnosis [1].

Pedicle screws for osteoporotic spines are not so effective because screw loosening, screw pullout, and implant construct failure are relatively common [2]. To solve these problems, several procedures have been reported, such as cement-augmented screws [3], specialized expandable screws [4], and transdiscal screws [5]. Each method has advantages and drawbacks, including bone cement-related complications and significantly increased implant cost. The new technique of triangular fixation was reported for adult spinal deformity surgery to prevent proximal screw backout [6]. The purpose of this report is to present our novel delta fixation technique for DISH-associated spinal fractures performed percutaneously under navigation.

## Case presentation

Patient history

An 86-year-old male was referred to our emergency department in September 2023 with severe back pain following a fall. The patient was alcoholic and had a history of gastric ulcers. He had slight back pain and complained of spinal stiffness for more than 20 years.

Physical examination

Due to severe low back pain, he could not walk or sit, and all attempted movements were limited. However, he showed no motor deficits or sensory disturbance. His deep tendon reflex was normal. There was percussion-associated pain in the lumbar area, and his visual analog scale for low back pain was 82/100 mm.

Preoperative imaging

**Figure 1:**
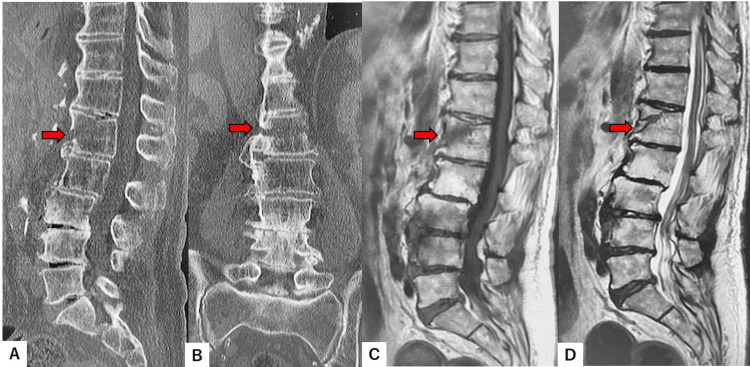
Preoperative images A: Mid-sagittal reconstruction CT. B: Coronal reconstruction CT. C: Mid-sagittal T1-weighted MRI. D: Mid-sagittal T2-weighted MRI. Red arrows indicate fracture line. CT: computed tomography, MRI: magnetic resonance imaging

Preoperative spinal reconstruction CT showed DISH and L1 fracture (AO type B3). There was a 5 mm gap in the L1 fracture site. MRI revealed hypointensity in the L1 vertebra, indicating acute fracture and no epidural hematoma or spinal cord compression at the L1 level (Figure 1).

Surgical technique

The patient was positioned in the prone position on a breaking Jackson frame to get a CT scan by O-arm. Care was taken not to open up the fracture site, as patients with ankylosed spines often have a kyphotic spine alignment. The procedure was performed under neuro-monitoring. The reference frame for the navigation is fixed at the iliac bone, the O-arm is then positioned, and the 3D reconstructed images are obtained and transmitted to the Stealth station navigation system Spine 7 R (Medtronic, Medtronic Sofamor Danek, Minneapolis, MN).

**Figure 2:**
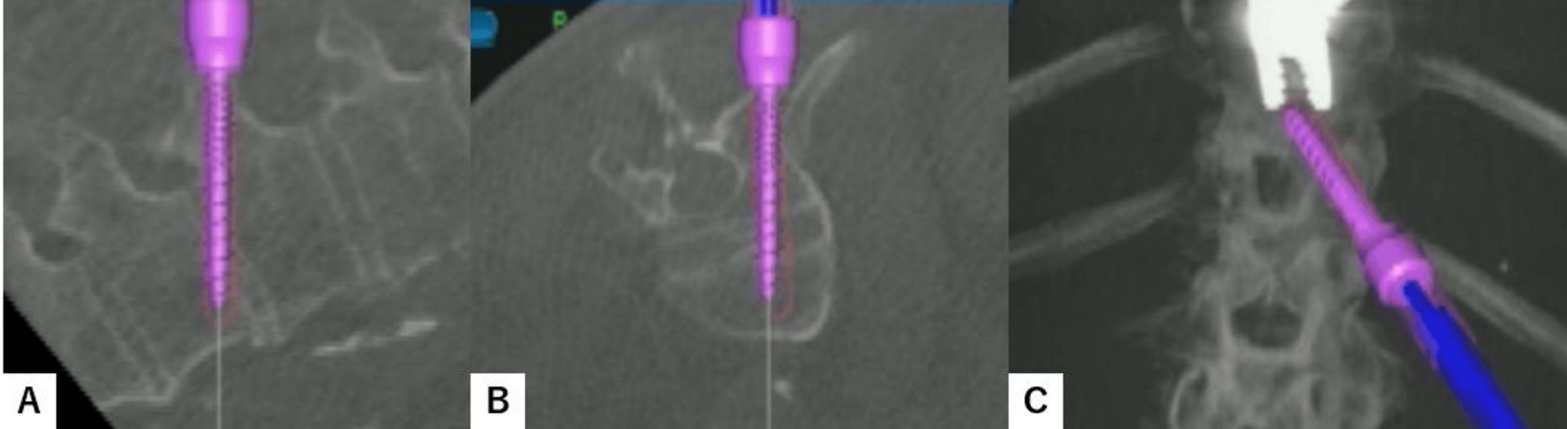
Transdiscal screw insertion A: Axial view. B: Sagittal view. C: Coronal view.

**Figure 3:**
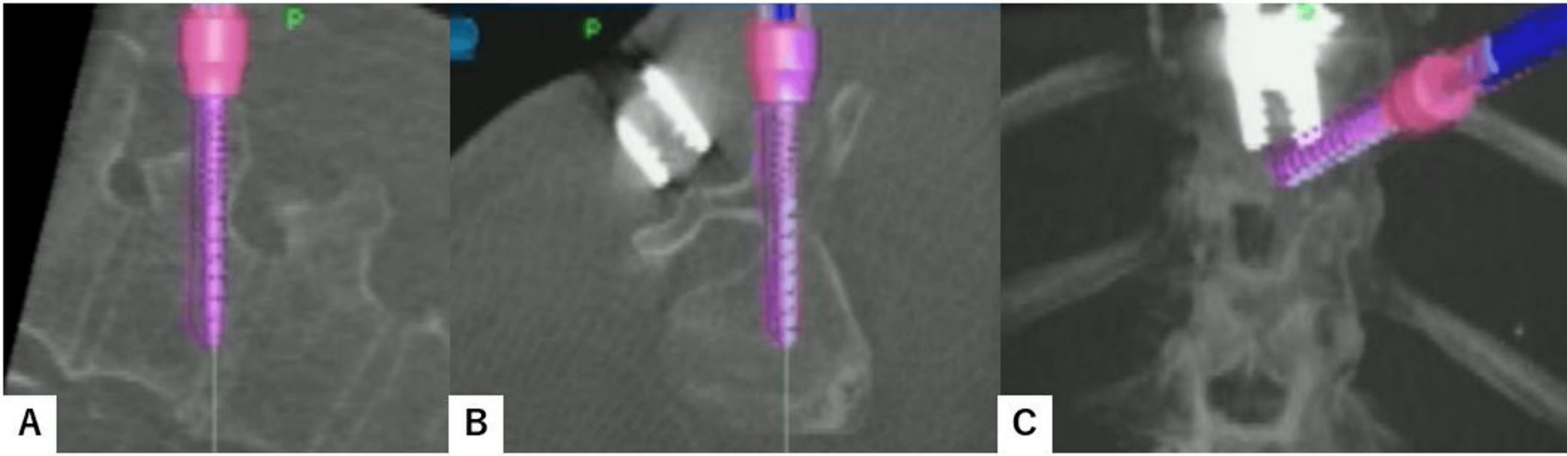
Downward screw insertion A: Axial view. B: Sagittal view. C: Coronal view.

After verifying every navigated spinal instrument, the best entry point for each screw was marked by the navigated pinpoint probe. Percutaneous transdiscal screws of T12 and L3 were aimed toward the upper endplate and directed approximately 25 degrees cranially to penetrate the superior endplate of the working level (Figure 2). Downward pedicle screws of T11 and L2 were aimed toward the lower and anterior end of the endplate to make a triangular shape on each side (Figure 3).

**Figure 4:**
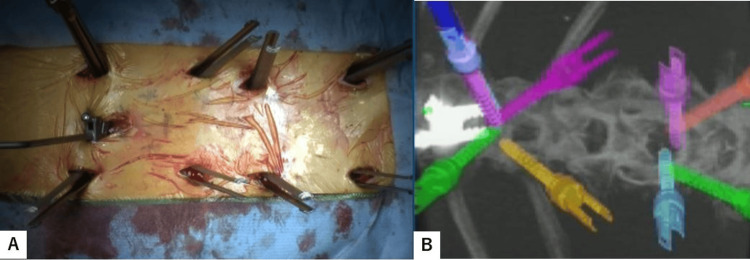
Intraoperative image and navigation image A: Intraoperative image. B: Navigation image.

For the rod and screw interface, more than 30 degrees screw tilt is not allowed because multiaxial percutaneous pedicle screws can be angulated only 30 degrees to the rod. Screw length and diameter are also measured using the navigation system. The screw pilot holes are then tapped. During screw insertion, an increase in the insertional torque could be appreciated when the screw was engaging the endplate. Intraoperative anteroposterior and lateral radiograms are obtained to check the correct placement of each screw (Figure 4).

The breaking Jackson frame should be bent convexly if the fracture gap is unacceptable. Then, appropriately contoured rods were inserted percutaneously and attached to the screw. Care is taken to make sure a gap at the fracture site is not inadvertently created when tightening the screws to the rods.

Postoperative images

**Figure 5:**
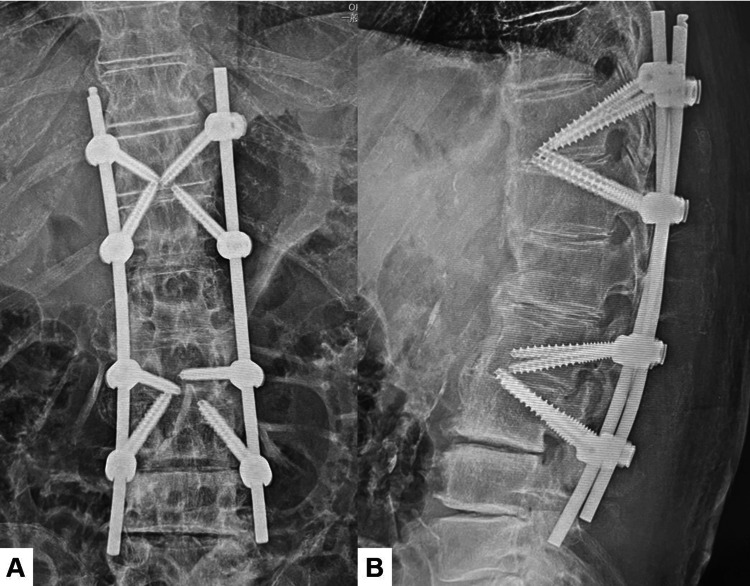
Postoperative radiograms A: Anteroposterior lumbar radiogram. B: Lateral lumbar radiogram. The screws' construct is triangular.

**Figure 6:**
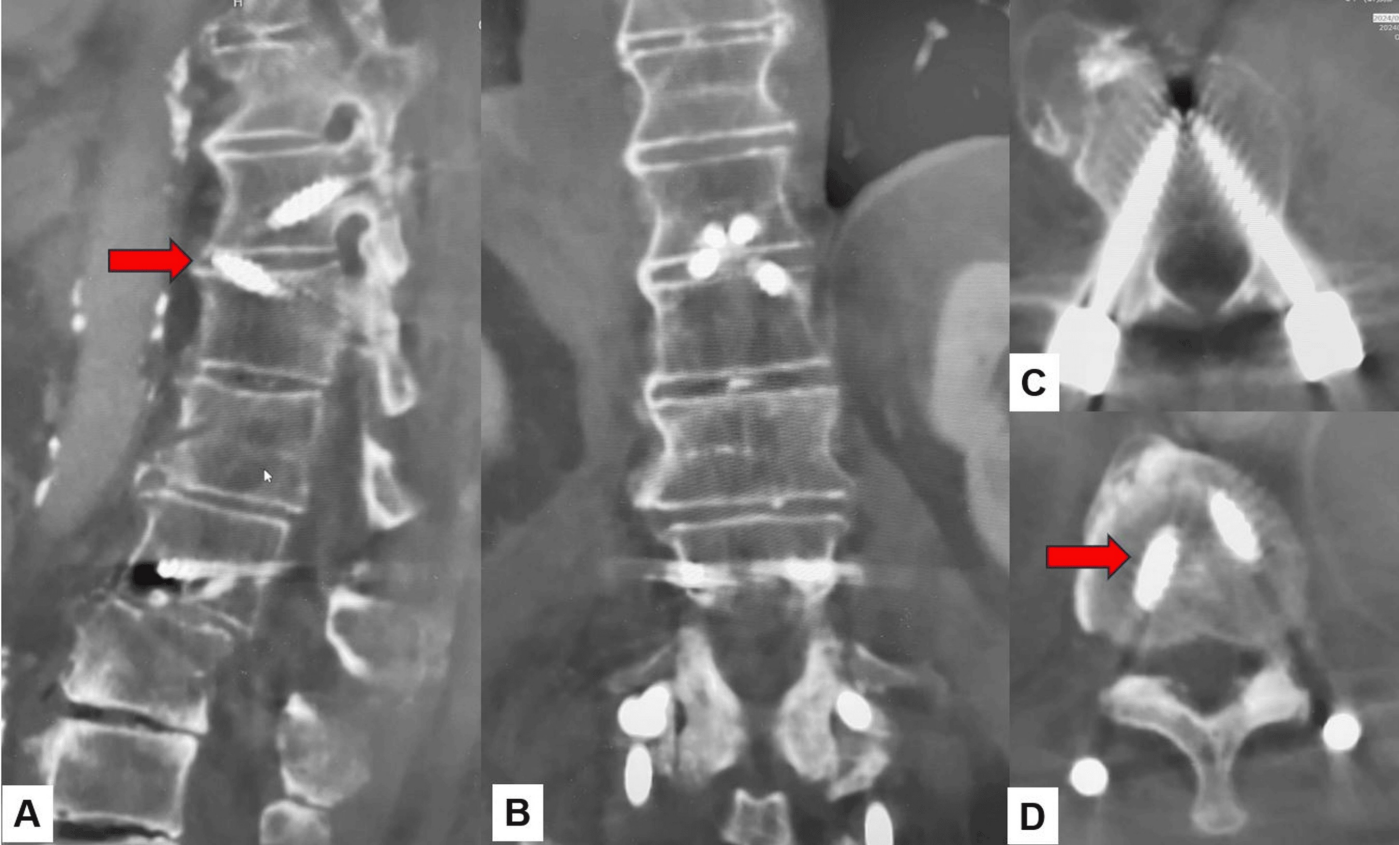
Postoperative CT A: Paramedian reconstruction CT. B: Coronal reconstruction CT. C: Axial CT at T11. D: Axial CT at L2. The transdiscal screws are penetrated into the endplate (red arrows). CT: computed tomography

Postoperative spinal radiograms showed good spinal alignment. In a lateral spinal radiogram (delta fixation), the upper and lower segments of the fracture site resembled two triangle shapes (Figure 5). Postoperative CT indicated that all screw positions were satisfactory (Figure 6).

Clinical results

One week after surgery, he could walk without support. He had no neurological deficit, and his visual analog scale for low back pain improved from 82 mm to 23 mm.

## Discussion

Diffuse idiopathic skeletal hyperostosis (DISH) is common among the elderly. Ikuma et al. reported a prevalence of 17.4% in the Japanese population [7]. DISH is characterized by calcification and ossification of spinal ligaments, particularly the anterior longitudinal ligament [8]. This condition causes rigidity in the spine and movement impairment and can lead to an increased risk of vertebral fractures, especially in those with osteoporosis [9]. The fused vertebrae act as long levers, concentrating mechanical stress at specific fracture-prone points. This combination of conditions presents significant challenges for treatment, especially regarding surgical intervention and screw placement [10-12]. In treating vertebral fractures in patients with both osteoporosis and DISH, conservative methods such as rest and bracing are often insufficient. As a result, rigid internal fixation and early mobilization using pedicle screws have become a standard treatment option for patients with DISH who suffer vertebral fractures [13].

The design and placement of pedicle screws in these patients are critical to successful outcomes. Research on screw designs has shown that dual-thread screws provide superior primary pullout strength compared to single-thread and mixed-thread screws [14]. Dual-thread screws, which feature more threads per unit length, increase the surface area in contact with the bone, enhancing stability and reducing the risk of screw loosening [14]. In addition, tapping before insertion also provides a better bone implant situation, reducing the incidence of loosening and pullout [10]. Biomechanical studies have confirmed that dual-thread screws offer better stability, particularly in osteoporotic bone [15]. Mixed-thread (cortical and cancellous) screws, on the other hand, showed a reduction in revision pullout strength, highlighting their limitations in situations where screw reinsertion or adjustment is necessary [16]. Moreover, recent advancements in intraoperative imaging and navigation systems have improved the accuracy of screw placement and reduced the risk of malposition and reoperation [17].

Studies have demonstrated that the prevalence of DISH in patients with osteoporotic vertebral fractures is substantial, with one study showing that 33.9% of vertebral fracture patients also had DISH [11]. In such cases, the rigidity of the spine due to DISH complicates the diagnosis and treatment of fractures. Vertebral fractures in DISH patients tend to occur more frequently at the thoracolumbar junction since the primary point of DISH localization is around T9 [12]. This translates into a mechanical fulcrum susceptible to injury at the transition zone [11].

**Figure 7:**
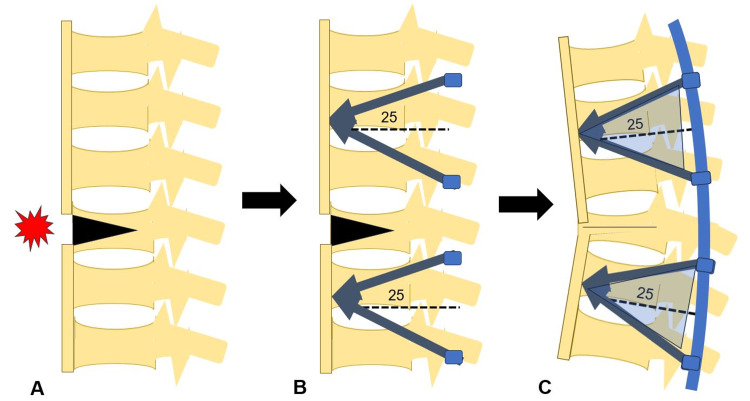
Step of delta fixation A: Preoperation. B: Screw insertion. C: Postoperation. Created by Masato Tanaka

In osteoporotic spines, screw placement poses a significant challenge due to the reduced bone mineral density, which increases the risk of screw loosening and pullout. To mitigate these risks, techniques regarding screw placement and the use of enhanced screw trajectories have been developed [7]. A biomechanical study by Shibasaki et al. showed that bicortical screws had significantly higher insertional torque and pullout strength compared to unicortical screws, particularly in the caudad trajectory, which aimed toward the anteroinferior corner of the vertebral body [18]. In traditional pedicle screw fixation, the screws may be at risk of loosening or pulling out due to the limited purchase of osteoporotic bone. The triangular screw placement technique, also known as delta fixation (Figure 7), offers a biomechanical advantage in preventing screw pullout, especially in patients with compromised bone quality, such as those with osteoporosis and DISH [19].

The concept involves using a minimally invasive approach and colocation of screws in a triangular arrangement guided by intraoperative navigation, with transdiscal screws placed cranially [19] on the inferior portion of every delta, combined with downward screws inserted into the vertebral body from the cephalic portion of each delta. This creates a quadruple triangular construct, significantly improving the fixation's stability. Additionally, this technique allows for better anchoring in osteoporotic bones. Studies have shown that triangular fixation significantly reduces the likelihood of screw pullout by providing multiple points of fixation across a broader area of the vertebra [19].

Therefore, combining the navigated tetra delta technique can provide several benefits in the surgical setting. First and foremost, the procedure's invasiveness is less than an open approach. It has been stated before that transdiscal screw offers 1.6-1.8 times the strength versus conventional transpedicular fixation, translating into fewer implants required to stabilize the segments compared with the usual three upper and three lower levels for osteoporotic fracture fixation [7,17,19]. There is a limitation of this method. Fluoroscopy alone may make accurate insertion difficult, and the use of navigation may be necessary to achieve precise screw placement.

## Conclusions

The combination of DISH and osteoporosis presents unique challenges in the treatment of vertebral fractures. The new minimally invasive delta fixation technique offers several advantages over traditional procedures, such as less tissue disruption by its minimally invasive nature. This novel technique also improved construct stability based on the principles of transdiscal screws and triangular screws spread in a four triangular construct base.
